# Gut microbiome diversity and composition is associated with exploratory behavior in a wild-caught songbird

**DOI:** 10.1186/s42523-023-00227-x

**Published:** 2023-02-04

**Authors:** Melanie R. Florkowski, Jessica L. Yorzinski

**Affiliations:** 1 Ecology and Evolutionary Biology Program, Texas A&M University, 534 John Kimbrough Blvd, College Station, TX 77843 USA; 2 Department of Ecology and Conservation Biology, Texas A&M University, College Station, TX USA

**Keywords:** House sparrow, *Passer domesticus*, Exploratory behavior, 16S rRNA gene, Gut microbiome, Biofilm formation, Xenobiotic degradation

## Abstract

**Background:**

The gut microbiome influences its host in a myriad of ways, from immune system development to nutrient utilization. However, our understanding of the relationship between the gut microbiome and behavior, especially in wild species, is still poor. One behavior that potentially interacts with the gut microbiome is exploratory behavior, which animals use to acquire new information from the environment. We hypothesized that diversity of the gut microbiome will be correlated with exploratory behavior in a wild-caught bird species. To test this hypothesis, we captured wild house sparrows (*Passer domesticus*) and collected fecal samples to measure the diversity of their gut microbiomes. We then introduced individuals to a novel environment and measured their exploratory behavior.

**Results:**

We found that birds with higher alpha diversity of the gut microbiome exhibited higher exploratory behavior. These results suggest that high exploratory birds encounter more types of environmental microbes that contribute to their diverse gut microbiome compared with less exploratory birds. Alternatively, increased gut microbiome diversity may contribute to increased exploratory behavior. We also found differences in beta diversity when comparing high and low exploring birds, indicating differences in microbiome community structure. When comparing predicted functional pathways of the birds’ microbiomes, we found that the microbiomes of high explorers contained more pathways involved in biofilm formation and xenobiotic degradation than those of low explorers.

**Conclusions:**

Overall, we found that the alpha and beta diversity of the gut microbiome is correlated with exploratory behavior of house sparrows. The predicted functions of the gut microbiome from high explorers differs from that of low explorers. Our study highlights the importance of considering the gut microbiome when investigating animal behavior.

**Supplementary Information:**

The online version contains supplementary material available at 10.1186/s42523-023-00227-x.

## Background

The gut microbiome of an animal in an essential part of its biology with effects ranging from nutrient absorption, immune status, and even cognition [1–3]. Despite recent increased attention to wild species, the majority of research on the microbiome has been in model organisms such as rodents and *Drosophila* [4–6]. The microbiome is likely an important factor in the fitness of wild organisms and therefore research into the structure and function of the microbiome of wild species is needed [7]. The gut microbiome varies between species, and it is also highly variable within individuals of the same species and throughout the life of an individual [8, 9]. Microbiome diversity can be crucial for normal functioning and a more diverse microbiome is generally considered more stable and provides health benefits to its hosts [10, 11]. Studies in laboratory animals have found that the diversity of the gut microbiome varies depending on a number of factors including host genetics, diet, and the surrounding environment [12–15]. Research on the microbiome is a rapidly growing field, however there is still much we do not understand, especially in wild-caught organisms.

One factor that can potentially influence the gut microbiome of wild organisms is exploratory behavior. Exploratory behavior is a key trait where animals learn new information about their environment [16]. Exploratory behavior informs how individuals interact with their environment and may be an important factor in determining which microorganisms are able to colonize a host. Individuals within a species typically vary in their exploratory behavior [19] and in addition to influencing animal fitness through altering predator exposure [17] and reproductive success [18] this variation may be an important factor in determining which microorganisms an individual is exposed to and can colonize their microbiome. Due to the importance of the microbiome, this may result in exploratory behavior impacting host fitness through the microbiome.

Alternatively, the gut microbiome may drive differences in exploratory behavior. For example, gnotobiotic mice exhibit increased exploratory behavior compared to mice with normal microbiomes due to altered gene expression in the brain [20]. Similarly, axenic *Drosophila* have increased locomotor activity attributed to the microbiome’s role in regulating the hormone octopamine [21]. Probiotic administration also increases exploratory behavior in mice by altering hormones and neurotransmitters [22, 23]. The interaction between behavior and the microbiome is likely interrelated, and we still have a limited understanding of how natural microbiomes relate to exploratory behavior.

In this study we evaluated the hypothesis that diversity of the gut microbiome will be correlated with exploratory behavior in a wild-caught bird species, the house sparrow (*Passer domesticus*). House sparrows have documented variation in exploratory behavior that has not yet been fully explained [26]. House sparrows are also an invasive species that now have a global distribution. Understanding the sparrow’s behavior is an important component for understanding this species’ success as an invader [27]. We predicted that birds exhibiting higher exploratory behavior would have higher alpha diversity, a measure of the richness and evenness of the microbiome. We also predicted there would be differences in beta diversity, a measure of the similarity between microbial communities, and functional pathways among individuals with different levels of exploratory behavior.

## Results

We measured the exploratory behavior of wild-caught house sparrows (*n* = 45) in a novel environment and quantified their gut microbiome with fecal samples collected at capture by sequencing the 16S rRNA gene. We also collected a second fecal sample from a subset of the bird (*n* = 31) after the novel environment test. For all samples collected, the sequencing output resulted in 2,656,466 raw reads. After quality control (which resulted in us omitting the samples of 5 pre-captivity samples and 1 post-captivity sample due to low read counts), we processed the 70 total fecal samples (40 pre-captivity and 30 post-captivity) with an average number of sequence reads 23,114 ± 13,337 (mean ± standard deviation) clustered into a total of 10,470 operation taxonomic units (OTUs).

**Fig. 1:**
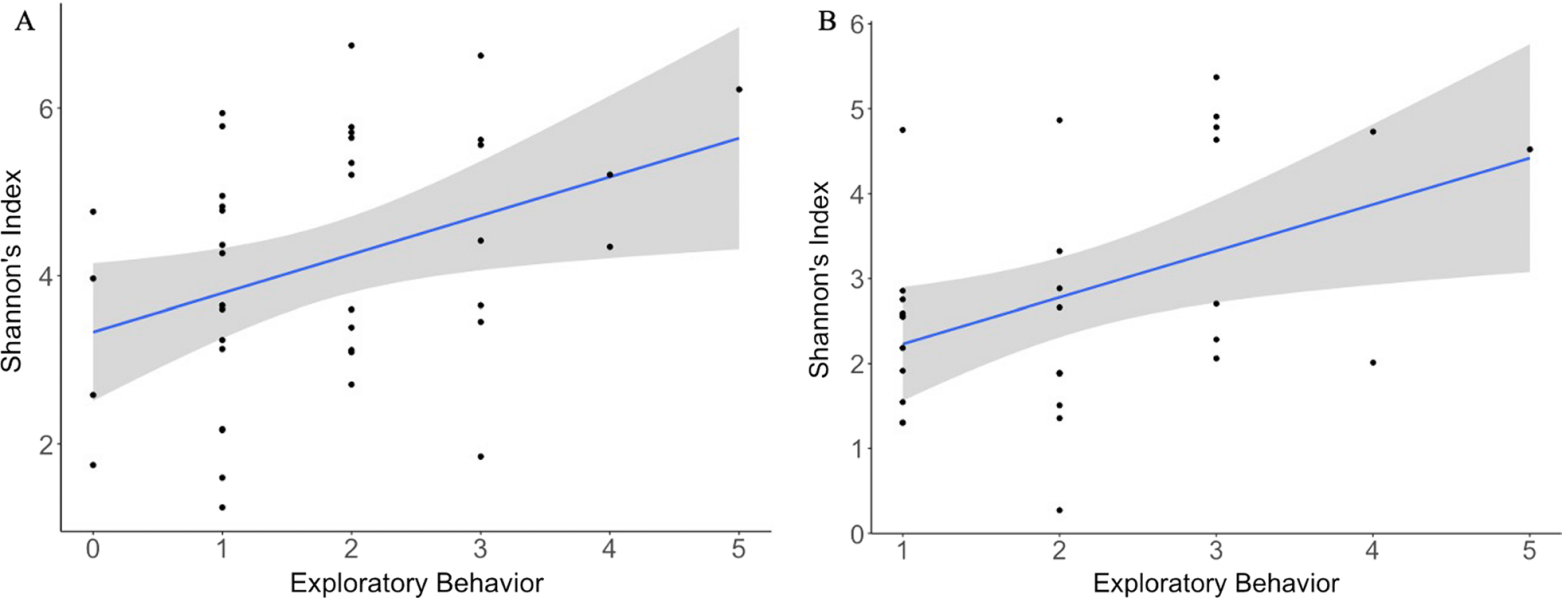
Relationship between exploratory behavior (number of objects visited) and Shannon diversity index of the gut microbiome of samples collected before (**A**) and after (**B**) captivity

We found that alpha diversity (measured with Shannon diversity index) of the microbiome community is positively correlated with exploratory behavior (Table 1; Fig. 1). Birds that had higher alpha diversity of their gut microbiome also had higher exploratory behavior. This result is robust to measuring alpha diversity using different methods and the same result is seen with Chao1 diversity index and observed OTUs (Additional file 1: Table S1).

**Table 1:** Results of linear mixed-effect model investigating the influence of exploratory behavior, sex, scaled mass, activity level and latency to explore on Shannon’s index of the gut microbiome of the birds collected before and after captivity

Variable	Degrees of freedom	Pre-captivity: F value (*P*-value)	Post-captivity: F value (*P*-value)
Exploratory Behavior	1	8.87 (0.009*)	9.00 (0.006*)
Sex	1	0.67 (0.421)	4.15 (0.053)
Scaled Mass	1	3.43 (0.074)	0.76 (0.391)
Activity Level	1	0.93 (0.342)	3.66 (0.068)
Latency to Approach First Object	1	0.28 (0.599)	0.66 (0.422)

* indicates significance at 0.05 level

**Fig. 2:**
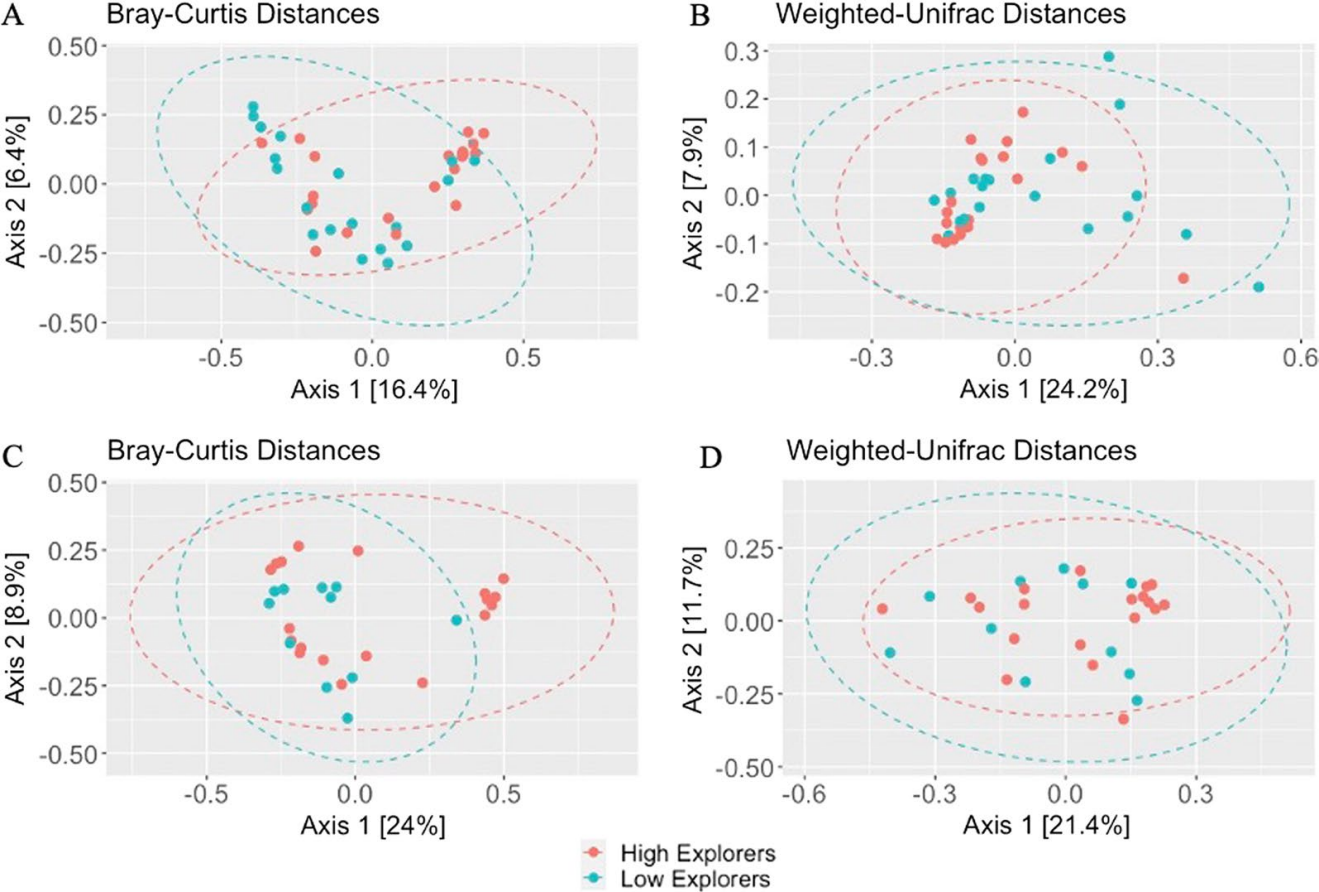
Principal coordinate analysis of pre-captivity (**A**) Bray–Curtis (**B**) weighted UniFrac distances and post-captivity (**C**) Bray–Curtis (**D**) weighted UniFrac distances between the gut microbiomes of high (red) and low (blue) explorer birds. Each point represents a different individual. Circles indicate 95% confidence intervals

We also found significant differences in beta diversity between high and low explorers using Bray–Curtis distances and near significant differences in weighted Unifrac distances in pre-captivity samples but not post-captivity samples (PERMANOVA, Fig. 2; Table 2). Principal components one and two explained 16.4% and 6.4%, 24% and 8.9%, 24.2% and 7.9%, and 21.4% and 11.7% of the variance in gut microbiome communities based on pre- and post-captivity Bray–Curtis and pre- and post-captivity weighted UniFrac distances, respectively.

**Table 2:** Results of PERMANOVA investigating the influence of exploratory group, sex, scaled mass, activity level, latency to explore, capture location, and capture date on differences in the birds’ pre-captivity and post-captivity gut microbiome communities using Bray–Curtis dissimilarity or Weighted UniFrac distance

Variable	Degrees of freedom	Pre-captivity Bray–Curtis: F value (*P*-value)	Post-captivity Bray–Curtis: F value (*P*-value)	Pre-captivity Weighted UniFrac: F value (*P*-value)	Post-captivity Weighted UniFrac: F value (*P*-value)
Exploratory group	1	1.62 (0.026*)	1.29 (0.154)	1.65 (0.054)	0.77 (0.762)
Sex	1	1.47 (0.058)	1.40 (0.112)	2.18 (0.023*)	1.56 (0.071)
Scaled mass	3	1.83 (0.014*)	1.34 (0.161)	2.82 (0.005*)	1.62 (0.069)
Activity levels	3	1.10 (0.278)	0.98 (0.441)	0.85 (0.608)	0.66 (0.885)
Latency to approach first object	3	0.81 (0.806)	0.84 (0.644)	0.95 (0.451)	0.73 (0.773)
Capture location	4	1.27 (0.038*)	1.31 (0.113)	1.45 (0.058)	1.02 (0.442)
Capture date	24	1.19 (0.030*)	1.29 (0.079)	0.98 (0.539)	1.01 (0.471)

* indicates significance at 0.05 level

There was significant heterogeneity in the dispersion of both Bray–Curtis (F_1,43_ = 9.4, *p* = 0.003) and weighted UniFrac (F_1,43_ = 4.1, *p* = 0.048) distances. However, PERMANOVA is robust to heterogeneity when the compared groups have similar samples sizes, therefore this was unlikely to bias results [40]. The only taxa differentially abundant between the high and low explorers was *Catellicoccus* (OTU 1), and it was more abundant in low explorers (LDA = 5.40, *p* = 0.0267).

**Fig. 3:**
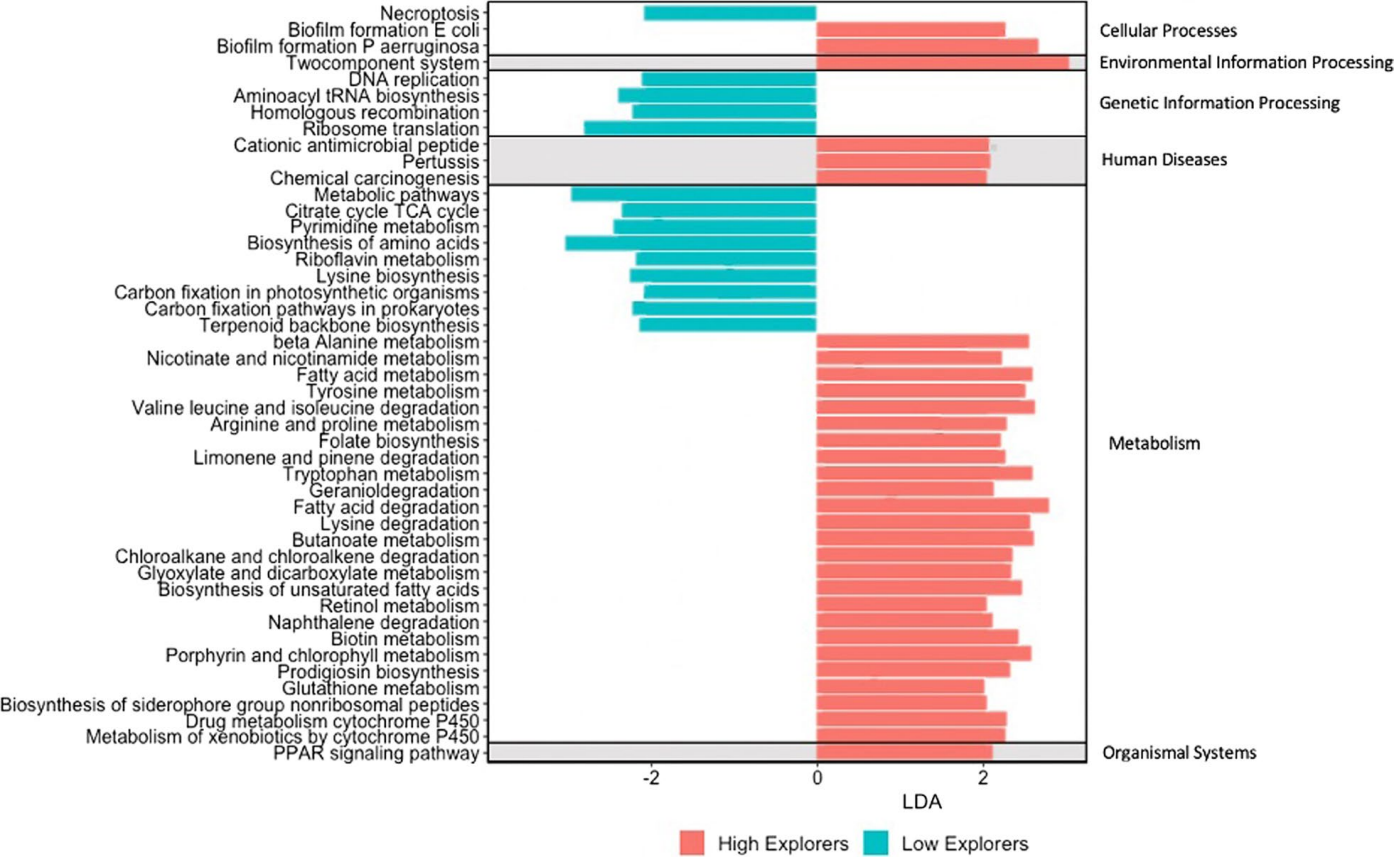
Linear Discriminant Analysis Effect Size results identifying functional pathways differentially abundant in the gut microbiomes of high and low explorers

The microbiome functional analysis predictions resulted in 368 predicted pathways defined by the Kyoto Encyclopedia of Genes and Genomes (KEGG) pathway database. Tax4Fun2 only utilizes sequences that have matches to the reference databases to predict functions and on average 85.9 ± 22.1% (mean ± standard deviation) of sequences per sample were utilized. Thirty-two pathways were significantly enriched in the high explorers and 14 pathways were significantly enriched in the low explorers. The majority of pathways with significantly different abundance between the high and low exploratory groups were related to metabolism (78% of pathways abundant in high explorers versus 64% pathways abundant in low explorers) with the rest being genetic information processing (0% of pathways abundant in high explorers versus 28.5% pathways abundant in low explorers), cellular processes (6.15% of pathways abundant in high explorers versus 7.14% pathways abundant in low explorers), human diseases (9.3% of pathways abundant in high explorers versus 0% pathways abundant in low explorers), environmental information processing (3.12% of pathways abundant in high explorers versus 0% pathways abundant in low explorers) and organismal systems (3.12% of pathways abundant in high explorers versus 0% pathways abundant in low explorers) (Fig. 3).

## Discussion

Our results indicate that exploratory behavior in wild-caught house sparrows is positively correlated with the alpha diversity of their gut microbiome. High explorers had higher diversity relative to low explorers. Beta diversity also differed between high and low explorers, indicating that high explorers had different microbiome compositions than low explorers, however, this was only the case in pre-captivity samples. Finally, the functional pathways present in the microbiomes of high and low explorers differed. These findings provide the first evidence that the microbiome community is related to host exploratory behavior in a wild-caught species.

Birds that were high explorers in our novel environment had high alpha diversity in their gut microbiomes both before and after time spent in captivity. It is likely that birds that were high explorers in our novel environment were also high explorers in the wild, as exploratory traits measured in the lab in other passerine species have been found to reflect behavior in the wild [41]. Because the gut is colonized with microorganisms present in the host’s environment [42–45], the high explorers likely encountered more diverse microorganisms within these varied habitats compared with low explorers. Host organisms encounter microorganisms from the environment through interacting with substrates, like soil and leaf litter, as well as by ingesting microorganisms on food [42, 46, 47]. For example, correlations between diet and specific bacteria taxa were found in an insectivorous passerine, attributed to exposure to different bacteria from different diets [48]. Furthermore, these high exploring birds potentially encountered more diverse microorganisms through social interactions. In another passerine, high explorers interact more with other individuals, potentially exposing them to diverse microorganisms through social contact [49, 50].

Other studies have also found evidence that habitat use influences microbiome diversity. Migratory birds that reside in different habitats throughout the year have a distinct microbiome composition from non-migrant individuals of the same species [51]. Rural gulls (*Larsus argentatus*) with higher habitat heterogeneity and lower site fidelity had higher microbiome diversity compared to more urban birds [52]. Global change and urbanization are currently presenting challenges to many species and have required rapid adaptation. These habitat changes also present challenges in terms of maintaining a diverse and stable microbiome, which may have detrimental effects on host health [53].

It is possible that the microbiome is influencing exploratory behavior rather than vice versa. Behavioral traits can be impacted by microbiome composition through mechanisms such as altered gene expression, hormones, and neurotransmitters [20, 23, 54, 55]. For example, gnotobiotic mice had higher exploratory behavior as well as altered gene expression in the brain [20]. Recent research in birds has also found that microbiome diversity and composition was correlated with behavior and performance in cognitive tasks [56, 57]. It is also possible that another variable is driving the correlation between exploratory behavior and microbiome composition. For example, an individual’s genotype contributes both to behavior [58, 59] and microbiome composition [60, 61] and recent work in mice have found complex interactions between genotype and microbiome resulting in changes in behavior [62]. Exploratory behavior and microbiome diversity are possibly interrelated and contribute to each other. Experimental studies are therefore needed to determine the causality of this relationship.

We also found significant differences in the beta diversity between high and low exploring birds. Therefore, high explorers not only have more diverse microbiome communities but have different composition compared to low explorers. In addition, beta diversity significantly differed based on scaled body mass, possibly because individuals with different body masses consume different diets or have different genotypes. There are likely other factors that are important in determining differences in the microbiome composition, as the pre-captivity samples had clustering in the PCoA analysis that is unexplained by any of our measured variables. Interestingly, beta diversity did not significantly differ in exploratory behavior or scaled mass in the post-captivity samples, possibly because the standard captive environment reduced the differences in the microbiome between high and low exploring birds and birds of different masses. Despite differences in beta diversity in pre-captivity samples, the only taxon that was found to be significantly different between the two groups is a *Catellicoccus* bacteria, a common genus in the guts of several bird species [63–66]. The structure of the *Catellicoccus marimammalium* genome, the first described species in this genus, indicates that it is a gut symbiont, however its role in the gut microbiome is still not well described [67].

To further understand the differences between the microbiome communities, we investigated differences in their functional pathways. We predicted that high and low explorers would differ in the functional capabilities of their microbial communities. In the high explorers, their gut microbiomes had higher numbers of functions related to the formation of biofilms by opportunistic pathogens and the metabolism of xenobiotics. Biofilm formation can be harmful to the host when initiated by opportunistic pathogens such as *Pseudomous aeruginosa* [68]. Birds with higher exploratory behavior (and higher numbers of functions related to the formation of biofilms) therefore may have immune systems that are able to suppress the formation of these biofilms within their guts [69]. Exploration and resistance to pathogens are both important traits in a successful invasive species such as the house sparrow [70, 71]. However, it is not known if the gut microbiome mediates the relationship between immunity and behavior in this species. Xenobiotics are environmental toxins and high explorer birds may have microbiomes that are better at degrading and eliminating ingested xenobiotics than low explorers. Similarly, urban house sparrows have microbiomes with higher levels of xenobiotic degrading genes compared with rural house sparrows [72]. Therefore, the more exploratory birds may be better adapted to these birds’ urban environment. In addition to these functions, we also found several other differences between these groups in metabolism, cellular processes (such as DNA replication and transcription), and pathways for responding to environmental conditions. All these functions are predictions and therefore direct measurement of the microbiome’s functional capabilities and experimental exploration on their impacts is needed to understand how it relates to the birds’ exploratory behavior.

## Conclusion

Our results show that exploratory behavior positively correlates with alpha diversity in the gut microbiome in a wild-caught bird species. We also found that beta diversity and some functional pathways are significantly different between levels of exploratory behavior. These results suggest that differences in behavior may be driving differences in microbiome diversity and composition by influencing the variety of microbe taxa able to colonize the host. It is also possible that differences in the microbiome are driving differences in exploratory behavior. The results of this study are correlational but provide a basis for further explorations on this topic. Interesting next steps could include experimental manipulations of the microbiome (via diet manipulations or antibiotics) to determine the impact on avian behavior as well as investigating the relationship between the microbiome and other behaviors (such as sociality).

## Methods

### Animals and housing

From March through July 2020, we captured 45 adult house sparrows from 5 capture sites (at least 0.5 km apart, 3.2 ± 1.9 km (mean ± standard deviation)) in College Station and Bryan, Texas. These locations consisted of residential areas (30^o^67’N, 96^o^32’W; 30^o^59’N, 96^o^32’W; 30^o^59’N, 96^o^32’W), a suburban park (30^o^62’N, 96^o^35’W), and a poultry research center (30^o^58’N, 96^o^35’W). Birds were caught in either potter traps or mist nets. Immediately after capture, birds were placed in a paper bag with a sterilized weightboat at the bottom to collect a fecal sample (methodology outlined in [24]. Fecal samples were collected as a proxy for the microbial community present in the gut [28, 29]. Once the birds had defecated (within 5 min), the feces were placed in an Eppendorf tube with a sterile spatula. Samples were placed on ice until they were able to be stored in a − 80 °C freezer (mean time to freezer 107 ± 63 min). Birds were then transported to the housing facility and individuals were marked with a numbered metal leg band. The weight and tarsus length of the birds were recorded and were used to calculate scaled mass index [25]. Birds were then housed in cages (0.6 m × 0.33 m × 0.3 m) at Texas A&M University, College Station Texas, USA (30^o^36’N, 96^o^21’W) in an indoor room (5 m × 6.3 m). Birds were housed alone in their cage in order to minimize microbe transfer between individuals, although they were in visual and auditory contact with other birds in the room. They were kept on a 13 h:11 h light:dark cycle at 24.0 ± 0.5 °C (mean ± standard deviation) provided with food (Royal Wing Wild Bird Food, Tractor Supply Co.) and water ad libitum*.* Birds were tested individually on the novel environment test (see below) within three days of capture (range: 1–3 days; mean ± standard deviation: 1.46 ± 0.64 days). We collected a second fecal sample from most of the birds (*n* = 31; using the same methods as above) after they completed the novel environment test (5–10 days post capture, average: 6.80 ± 1.72 days (mean ± standard deviation)).

### Novel environment test

The novel environment test was based on a previous design testing exploratory behavior in house sparrows [26]. We modified the design slightly (reduced the size of the novel environment and changed a few of the objects) to make it suitable for our laboratory space. The novel environment was an indoor flight cage (2 m × 2 m × 2 m) with sides and ceiling made of wire mesh. Inside the novel environment were nine objects that the bird could visit: two artificial trees, a nest shaped bag on the wall, a nest box, a food bowl, a perch, a bag hanging from the ceiling, a shelf, and a toy ball on the ground (Additional file 1: Figure S1). The experimenter placed each bird individually in the novel environment and its behavior was recorded for 30 min using two video cameras (VIXIA HF R70; Canon Inc.). The experimenter moved to an adjacent room during the trial and was not visible to the bird. After 30 min, the bird was removed from the flight cage and returned to its housing cage. Each bird was only tested once.

The videos from each trial were scored to record exploratory behavior and activity level. Exploratory behavior was measured by recording the number of different objects the bird physically contacted during the trial. Only the first instance of contact to an object was recorded if the bird visited an object multiple times, which occurred in most trials. Activity level was recorded by measuring the amount of time the bird spent moving between locations. The start of each movement was defined when the bird moved more than one body length from its current location and ended when the bird first contacted its next location and remained there for at least one second. We also recorded the latency to explore, which was the amount of time between the placement of the bird in environment and when it first contacted the first object in the flight cage. Videos were scored by two coders. To ensure reliability between video coders, the coders practiced on one trial, and they scored behaviors similarly (within three seconds of each other in all cases).

### Microbiome methods

We isolated DNA from 0.25 g of each fecal sample using QIAamp PowerFecal DNA Isolation Kits (Qiagen, Germany) following the manufacturer’s protocol (except we increased sample incubation at 65 °C from 10 min to at least 8 h in an effort to increase DNA yield). We used a Qubit fluorometer (dsDNA HS Assay Kit, Invitrogen, Carlsbad, United States) to verify sufficient DNA yield and dilute the sample to a concentration of 5 ng/ul of DNA. The extracted DNA was then sent to the Michigan State University’s genomics core and was processed and sequenced according to Kozich et al. [30]. In brief, libraries were constructed by amplifying the V4 region of the 16S rRNA gene using primers 515F and 806R with Illumina adapters and dual indices. Samples were amplified using DreamTaq Master Mix (ThermoFisher). The PCR reaction was incubated at 95 °C for 3 min, followed by 30 cycles of 45 s at 95 °C, 60 s at 50 °C, and 90 s at 72 °C, then a final extension at 72 °C for 10 min. PCR products were then pooled and were batch normalized using Invitrogen SequalPrep DNA Normalization plates. Products recovered from the plates were concentrated using an Amicon Ultra centrifugal filter and cleaned using AMPure XP magnetic SPRI beads. The cleaned pools were sequenced on the Illumina MiSeq platform using v2 2 × 250 base pair kit (Illumina, Inc).

Initial quality control of raw sequences was performed with *Trim Galore* (version 0.6.6) which was used to remove adaptors and trim reads with base quality below a Phred score of 20 resulting in 2,300,451 reads. Trimmed sequences were then processed using the *Mothur* software (version 1.45.3; [31] using standard operating procedure (accessed May 2021). Briefly, the sequences were assembled into contigs and further quality trimmed. Identical sequences were merged, and singletons were removed. Remaining sequences were aligned against the SILVA database (Release 132). Chimeric sequences were removed using the UCHIME function. Remaining sequences were clustered into Operational Taxonomic Units (OTUs) with 97% similarity. Samples with fewer than 1000 reads were dropped from subsequent analysis as the low sequence read count may skew downstream analysis [32].

### Statistical analysis

Files were imported into RStudio (version 4.1.2) using the *phyloseq* R package (version 1.36.0 [33]). Variance stabilization transformation was applied to the OTU counts to account for the differences in library size across samples using the *Deseq2* R package (version 1.32.0; [34]. Analyses were also performed using raw data and data rarified to 1810 sequences, which is size of the sample with the lowest read count after filtering, however, the analyses of these data yielded similar results, so we present them in the supplement only (Additional file 1: Tables S2–S5).

To quantify alpha diversity, we calculated Shannon’s index, Chao1, and observed OTUs (using *phyloseq* R package), which were then used as the dependent variable in linear mixed effect models. Chao1 and observed OTUs were log-transformed to better fit model assumptions. The independent variables of each model were the number of different objects the bird visited, scaled body mass, sex, latency to approach first object, and activity level. Capture location and date were used as the random effects in the model (*lme4* R package version 1.1–27.1; [35]).

To quantify beta diversity, which quantifies differences in microbial community composition, we divided birds into ‘high explorer’ (visited 2–5 objects; *n* = 25), and ‘low explorer’ (visited 0–1 objects; *n* = 20) groups. We then calculated dissimilarity matrices using both Bray–Curtis and weighted-UniFrac distances. We then used the adonis2 function to run PERMANOVA in the *vegan* R package (version 2.5–7; [36] set at 999 permutations, which compares the centroids of microbial communities of different groups with either Bray–Curtis or weighted UniFrac distances as the dependent variable. The independent variables included were exploratory behavior (high explorer or low explorer), sex, scaled body mass, activity levels, latency to visit first object, capture location and trial date. Date and capture location were included as fixed effects because PERMANOVA cannot accommodate mixed effect models. PERMANOVA also cannot accommodate continuous variables, so scaled body mass, activity levels, and latency to visit first object were converted into discrete variables by using quartiles to split data into four groups. We also compared the dispersion of the two groups with a PERMDIST test using the betadisper function in *vegan* [37]. We used principal coordinate analysis (PCoA) plots to visualize the dissimilarity distances between the groups. Finally, to determine any taxa that were significantly different between high and low explorers we used the Linear discriminate analysis (LDA) effect size (LEfSe) function in *Mothur*. The results from the analysis were adjusted for multiple comparisons with the Benjamini–Hochberg correction [38].

To generate predictions about the functions of the microbiome communities we used the *Tax4Fun2* R package (version 1.1.5) which calculates a functional profile by associating OTUs with KEGG orthologue functional genes (KO) and their metabolic pathways. Although *Tax4Fun2* does not measure functional capacity directly, it is considered more accurate at determining microbial functions than other programs [39]. We used the LEfSe function on the Galaxy server (http://huttenhower.org/galaxy) to determine which functional pathways are differentially abundant between the high and low explorers. The results from the analysis were adjusted for multiple comparisons with the Benjamini–Hochberg correction [38].

## Supplementary Information

**Additional file 1.** Figure S1 and Tables S1–S5 providing additional details on the novel enviornment design and reporting statistical results and supplementary microbiome analyses.

## Data Availability

Sequences analyzed in the current study were deposited in the NCBI database under BioProject PRJNA838556 (Accession no. SRX15281955—SRX15282029).
