# Supplementary material for: Gut microbiome diversity and composition is associated with exploratory behavior in a wild-caught songbird
Source: Anim Microbiome. 2023 Feb 4;5:8. doi: 10.1186/s42523-023-00227-x (PMC9899379; doi:10.1186/s42523-023-00227-x)
Supplement: Supplementary file 1 — Additional file 1. Figure S1 and Tables S1–S5 providing additional details on the novel enviornment design and reporting statistical results and supplementary microbiome analyses. [file 42523_2023_227_MOESM1_ESM.docx]

Supplement

A


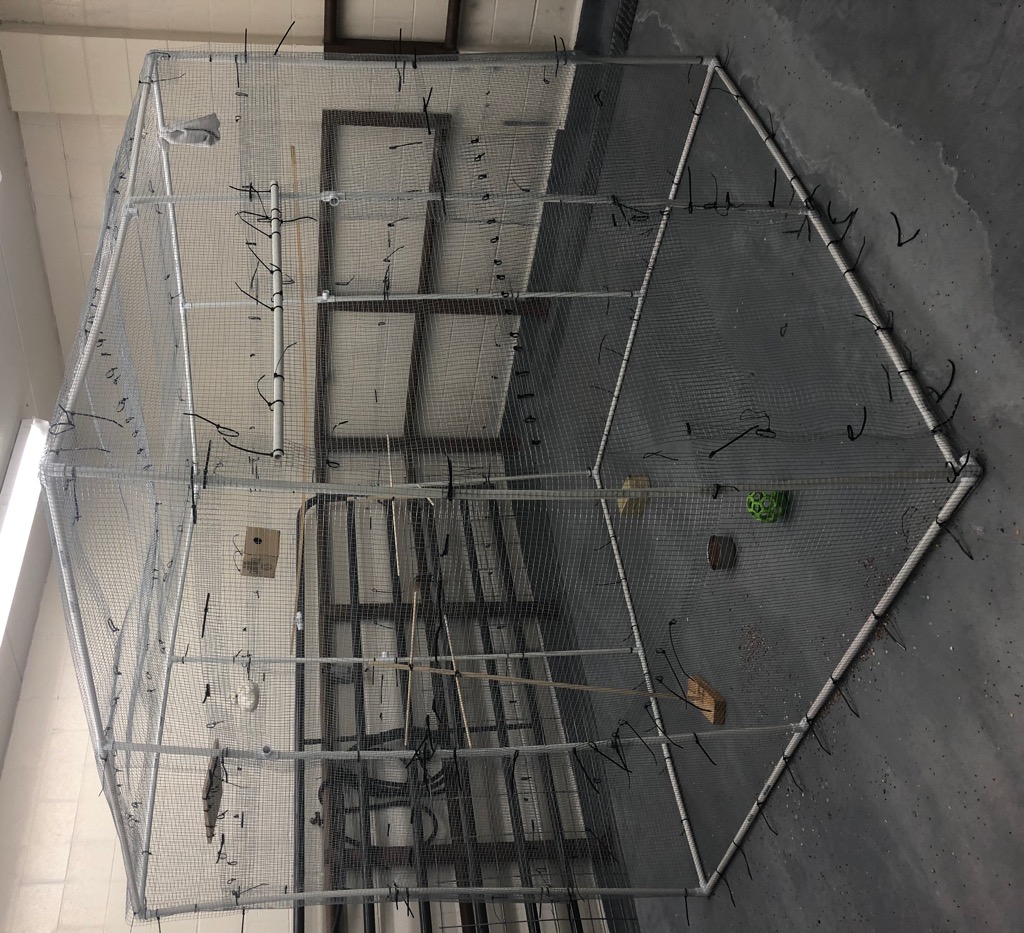


B

9

1

8

2

6

7

5

3

4

Figure S1. (A) Photo of exploratory chamber. (B) Schematic of exploratory chamber. (1) cardboard nest box, (2) wooden artificial tree (height of 130 cm), (3) cotton bag on ceiling, (4) green ball, (5) wooden perch, (6) food bowl with seeds inside, (7) wooden shelf, (8) wooden artificial tree (height of 130 cm), and (9) cotton bag on wall.

Table S1. Results of linear mixed-effect model investigating the influence of exploratory behavior, sex, scaled mass, activity level and latency to explore on Chao1 diversity index and observed OTUs of the gut microbiome of the birds collected before and after captivity.

| Variable | Degrees of  freedom | Chao1  Pre-captivity:  F value (P-value) | Chao1  Post-captivity:  F value (P-value) | Observed OTUs  Pre-captivity:  F value (P-value) | Observed OTUs  Post-captivity:  F value (P-value) |
| --- | --- | --- | --- | --- | --- |
| Exploratory  Behavior | 1 | 8.74 (0.011*) | 9.12 (0.006*) | 8.74 (0.009*) | 9.40 (0.005*) |
| Sex | 1 | 1.57 (0.229) | 5.19 (0.032*) | 0.75 (0.395) | 4.45 (0.045*) |
| Scaled Mass | 1 | 11.5 (0.002*) | 0.95 (0.338) | 3.86 (0.059) | 0.79 (0.381) |
| Activity Level | 1 | 1.12 (0.299) | 4.74 (0.039*) | 0.97 (0.332) | 4.08 (0.054) |
| Latency to Approach  First Object | 1 | 0.00 (0.974) | 1.01 (0.323) | 0.19 (0.662) | 0.69 (0.411) |

Table S2. Results of linear model investigating the influence of exploratory behavior, sex, scaled mass, activity level and latency to explore on Shannon’s index, log-transformed Chao1, and log-transformed observed OTUs using raw sequencing data collected before and after captivity.

| Variable | Degrees of  freedom | Shannon  Pre-captivity:  F value (P-value) | Shannon  Post-captivity:  F value (P-value) | Chao1  Pre-captivity:  F value (P-value) | Chao1  Post-captivity:  F value (P-value) | Observed OTUs  Pre-captivity:  F value (P-value) | Observed OTUs  Post-captivity:  F value (P-value) |
| --- | --- | --- | --- | --- | --- | --- | --- |
| Exploratory  Behavior | 1 | 5.76 (0.023*) | 8.67 (0.007*) | 2.26 (0.143) | 0.01  (0.921) | 2.31 (0.139) | 0.02 (0.883) |
| Sex | 1 | 1.89 (0.179) | 2.27 (0.146) | 1.59 (0.085) | 6.80 (0.015*) | 1.68 (0.203) | 6.65 (0.016*) |
| Scaled Mass | 1 | 1.54 (0.224) | 0.95 (0.795) | 0.06 (0.059) | 0.06 (0.807) | 3.10 (0.088) | 0.05 (0.810) |
| Activity Level | 1 | 1.56 (0.221) | 0.48  (0.494) | 1.74 (0.196) | 2.70 (0.114) | 1.74 (0.197) | 2.65 (0.117) |
| Latency to Approach  First Object | 1 | 1.04 (0.315) | 1.66 (0.213) | 0.14 (0.708) | 0.06 (0.805) | 0.14 (0.709) | 0.06 (0.800) |

Table S3. Results of PERMANOVA investigating the influence of exploratory group, sex, scaled mass, activity level, latency to explore, capture location, and capture date on differences in the birds’ pre-captivity and post-captivity gut microbiome communities using Bray-Curtis dissimilarity or Weighted UniFrac distance using raw sequencing data.

| Variable | Degrees of freedom | Pre-captivity Bray-Curtis:  F value (P-value) | Post-captivity Bray-Curtis:  F value (P-value) | Pre-captivity Weighted UniFrac:  F value (P-value) | Post-captivity Weighted UniFrac:  F value (P-value) |
| --- | --- | --- | --- | --- | --- |
| Exploratory Group | 1 | 2.09 (0.042*) | 2.04 (0.078) | 1.12 (0.311) | 1.38 (0.206) |
| Sex | 1 | 0.57 (0.836) | 0.96 (0.430) | 0.71 (0.722) | 0.94 (0.438) |
| Scaled Mass | 1 | 0.97 (0.440) | 0.91 (0.455) | 1.68 (0.080) | 1.54 (0.139) |
| Activity Levels | 1 | 0.84 (0.560) | 0.85 (0.493) | 0.64 (0.795) | 0.65 (0.745) |
| Latency to Approach First Object | 1 | 0.52 (0.887) | 0.84 (0.533) | 0.58 (0.874) | 0.73 (0.627) |
| Capture Location | 4 | 0.89 (0.580) | 1.81 (0.075) | 0.89 (0.656) | 0.91 (0.601) |
| Capture Date | 24 | 1.11 (0.305) | 2.05 (0.013*) | 0.91 (0.731) | 1.15 (0.243) |

Table S4. Results of linear mixed effect model investigating the influence of exploratory behavior, sex, scaled mass, activity level and latency to explore on Shannon’s index, log-transformed Chao1, and log-transformed observed OTUs using data rarified to 1810 sequences, which is size of the sample with the lowest read count collected before and after captivity. =

| Variable | Degrees of  freedom | Shannon  Pre-captivity:  F value (P-value) | Shannon  Post-captivity:  F value (P-value) | Chao1  Pre-captivity:  F value (P-value) | Chao1  Post-captivity:  F value (P-value) | Observed OTUs  Pre-captivity:  F value (P-value) | Observed OTUs  Post-captivity:  F value (P-value) |
| --- | --- | --- | --- | --- | --- | --- | --- |
| Exploratory  Behavior | 1 | 8.67 (0.007*) | 8.95 (0.006*) | 0.01 (0.921) | 3.78 (0.065) | 0.02 (0.883) | 14.9 (0.001*) |
| Sex | 1 | 2.27 (0.146) | 2.20 (0.152) | 6.80 (0.015*) | 6.98  (0.014*) | 6.65 (0.016*) | 6.14 (0.020*) |
| Scaled Mass | 1 | 0.06 (0.795) | 0.11 (0.737) | 0.06 (0.807) | 0.14 (0.702) | 0.05 (0.810) | 0.01 (0.945) |
| Activity Level | 1 | 0.48 (0.494) | 0.48 (0.491) | 2.70 (0.114) | 0.17 (0.678) | 2.65 (0.117) | 1.06 (0.312) |
| Latency to Approach  First Object | 1 | 1.66 (0.213) | 1.54 (0.229) | 0.06 (0.805) | 1.58 (0.219) | 0.06 (0.800) | 0.04 (0.835) |

Table S5. Results of PERMANOVA investigating the influence of exploratory group, sex, scaled mass, activity level, latency to explore, capture location, and capture date on differences in the birds’ pre-captivity and post-captivity gut microbiome communities using Bray-Curtis dissimilarity or Weighted UniFrac distance using data rarified to 1810 sequences, which is size of the sample with the lowest read count.

| Variable | Degrees of freedom | Pre-captivity Bray-Curtis:  F value (P-value) | Post-captivity Bray-Curtis:  F value (P-value) | Pre-captivity Weighted UniFrac:  F value (P-value) | Post-captivity Weighted UniFrac:  F value (P-value) |
| --- | --- | --- | --- | --- | --- |
| Exploratory Group | 1 | 2.53 (0.032*) | 2.86 (0.041*) | 1.07 (0.352) | 1.35 (0.182) |
| Sex | 1 | 0.56 (0.802) | 1.33 (0.245) | 0.61 (0.831) | 0.87 (0.532) |
| Scaled Mass | 1 | 1.42 (0.160) | 0.57 (0.713) | 1.67 (0.087) | 1.56 (0.134) |
| Activity Levels | 1 | 0.53 (0.849) | 0.93 (0.432) | 0.57 (0.882) | 0.65 (0.776) |
| Latency to Approach First Object | 1 | 0.51 (0.821) | 0.90 (0.478) | 0.60 (0.859) | 0.75 (0.660) |
| Capture Location | 4 | 0.89 (0.564) | 2.27 (0.059) | 0.87 (0.696) | 0.91 (0.616) |
| Capture Date | 24 | 1.37 (0.122) | 2.56 (0.015*) | 0.90 (0.742) | 1.19 (0.164) |
